# High-throughput full-automatic synchrotron-based tomographic microscopy

**DOI:** 10.1107/S0909049510047370

**Published:** 2011-01-20

**Authors:** Kevin Mader, Federica Marone, Christoph Hintermüller, Gordan Mikuljan, Andreas Isenegger, Marco Stampanoni

**Affiliations:** a Swiss Light Source, Paul Scherrer Institute, Villigen, Switzerland; b Institute for Biomedical Engineering, Swiss Federal Institute of Technology and University of Zurich, Zurich, Switzerland

**Keywords:** tomography, automation, high-throughput, image processing, alignment

## Abstract

At the TOMCAT (TOmographic Microscopy and Coherent rAdiology experimenTs) beamline of the Swiss Light Source with an energy range of 8–45 keV and voxel size from 0.37 µm to 7.4 µm, full tomographic datasets are typically acquired in 5 to 10 min. To exploit the speed of the system and enable high-throughput studies to be performed in a fully automatic manner, a package of automation tools has been developed. The samples are automatically exchanged, aligned, moved to the correct region of interest, and scanned. This task is accomplished through the coordination of Python scripts, a robot-based sample-exchange system, sample positioning motors and a CCD camera. The tools are suited for any samples that can be mounted on a standard SEM stub, and require no specific environmental conditions. Up to 60 samples can be analyzed at a time without user intervention. The throughput of the system is dependent on resolution, energy and sample size, but rates of four samples per hour have been achieved with 0.74 µm voxel size at 17.5 keV. The maximum intervention-free scanning time is theoretically unlimited, and in practice experiments have been running unattended as long as 53 h (the average beam time allocation at TOMCAT is 48 h per user). The system is the first fully automated high-throughput tomography station: mounting samples, finding regions of interest, scanning and reconstructing can be performed without user intervention. The system also includes many features which accelerate and simplify the process of tomographic microscopy.

## Abstract 2

The automatization tools for high-throughput tomographic microscopy developed at the TOMCAT beamline of the Swiss Light Source are described

## 1. Introduction

Recent advances at the TOMCAT (TOmographic Microscopy and Coherent rAdiology experimenTs) beamline (Stampanoni *et al.*, 2007 ▶; Marone *et al.*, 2008 ▶; Hintermüller *et al.*, 2010 ▶) at the Swiss Light Source at the Paul Scherrer Institute (Villigen, Switzerland) have enabled high-quality tomographic scans to be completed in 3–15 min making high-throughput studies a reality. Although equipment control, sample adjustments and data acquisition at the beamline are straightforward and very user friendly, human intervention is nonetheless required for every single scan. In fact, for each scan the user has to enter the experimental area, which is locked and alarmed when the beam is on during data acquisition, change the sample, lock the experimental area, align the sample and start a tomographic scan. This procedure has several disadvantages. First, standard security measures currently in place at synchrotrons prescribe a sequence of safety procedures every time the measurement area is made accessible for human entry. These measures are mandatory and play an important role in safety at the beamline, but they are time-consuming and hinder exchanging samples in less than 5 min. This means that the time needed for security procedures required to enter the experimental area and manually change the samples can be longer than the acquisition time itself. In addition, outdated fully manual operation at TOMCAT involves mounting the samples directly on the rotation stage by screwing the sample or holder into a post on this stage. This operation requires the user to work close to sensitive components (*e.g.* rotation stage and scintillator screen), introducing the possibility of equipment damage and being torqued out of alignment, potentially resulting in beam-time loss for repair and re-alignment, or simply lower quality images. Furthermore, the task of sample alignment is in many cases a relatively straightforward methodical procedure; albeit, for every sample it is still done by hand, requiring the user’s active presence for the entire duration of the beam time. It is additionally prone to user error; especially for longer and overnight beam-time sessions. For some studies it is important that the sample is oriented in a specific or at least consistent manner. This is conventionally done by manually mounting the sample using a microscope-based set-up and manually ensuring that the sample is aligned properly. This appears to be sufficiently accurate, but it is quite time-consuming and highly sensitive to human error. The complete sample alignment system at TOMCAT involves the interplay between a robot-based exchange system, an X-ray projection-based sample region of interest and orientation system, and a user-adjustable tomography scan system.

To overcome user-based limitations, avoid unnecessary intervention and therefore possible mistakes, fully exploit the acquisition speed for high-throughput experiments and enable user-free operation at TOMCAT, we have developed a robot-based automatic endstation.

Other beamlines and tube-based X-ray scanners have implemented automated systems for sample management and exchange; specifically, the tomography beamline of 2-BM at the Advanced Photon Source (De Carlo *et al.*, 2006 ▶; Wang *et al.*, 2001 ▶; De Carlo, 2010 ▶), several protein crystallography and diffraction beamlines (Olieric *et al.*, 2009 ▶; Wang *et al.*, 2008 ▶; De Carlo & Tieman, 2004 ▶; Yamamoto *et al.*, 2005 ▶; Alzari *et al.*, 2006 ▶; Moser *et al.*, 2005 ▶), and some SCANCO µCT scanners (SCANCO Medical AG, 2009 ▶).

The most comparable set-ups at other tomography beamlines provide some automation steps (De Carlo, 2010 ▶), but none provide automation from region-of-interest detection to reconstructed datasets as described here.

While many of these existing implementations automate various tasks and aspects of tomographic imaging and crystallography, and are in several cases better optimized for specific experiments, none currently has the tools for complex region and sample detection or provide the completeness of automation from sample mounting to reconstructed datasets.

Additionally in many experiments, the angular orientation of the sample is quite important for two reasons. First, some samples do not easily fit into the field of view if they are not aligned vertically. Second, for some samples the data must be compared with previously measured data which may have a preferred orientation as in the case of mechanical testing in bone (Voide *et al.*, 2008 ▶). So if these results are to be compared, the samples must be aligned. While digital methods for this purpose exist, it simplifies reconstruction and data management if the data are measured already in the correct orientation.

These tools are mandatory for instance in genome-scale projects, commonly involving over 1000 samples, where the ability to consistently identify the same region in a large number of samples that can vary greatly in size is required. To undertake such a project at TOMCAT, we developed a workflow to smoothly and automatically manage the above-mentioned steps of the acquisition process. In particular, we implemented an automatic sample-exchange robot-based system (§2), designed an automatic alignment tool (§3), as well as a sequencer tool to run full experiments without intervention (§4). This workflow also facilitates time-evolution studies, where sample changes over the course of heating, cooling, drying and other similar processes are monitored with an extremely high degree of precision, as shown in §5.1.

## 2. Automatic sample exchange

We have developed an automatic sample-exchange robot-based system, which is tightly integrated through a graphical user interface (GUI) with the stage and measurement controls to coordinate sample positioning. Currently, the system allows for automated exchange of 60 samples, for which regions of interest can be interactively selected using live X-ray projections of the sample. Using these projections the user is able to reposition the sample until the correct region of interest is within the field of view and store these positions prior to batch measurement. With the same approach, samples can also be pre-aligned. These selected regions (multiple regions per sample are possible) will then be measured in a user-definable sequence (§4).

Experimental Physics Industrial Control System (EPICS) (Dalesio *et al.*, 1994 ▶) is the standard communication framework for beamline and external equipment control at the Swiss Light Source (SLS) and many other synchrotrons and large research facilities. The tools developed here are entirely integrated within EPICS so that future enhancements of the beamline will work seamlessly with the existing automation tools, and users wishing to control other aspects of the beamline during an automated experiment are able to do so.

**Figure 1:**
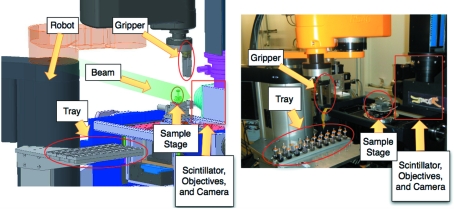
(Left) Three-dimensional rendering and (right) photograph of the robot, stage, beam and camera showing the layout of the automated end-station. The samples are picked up from the tray by the robot. The stage is then moved to a loading position safely away from the optical components. The robot then places the sample on the stage which is finally moved into the imaging position.

The sample exchange (robot) is a Stäubli 4-Axis RS40 system (Stäubli, Pfäffikon, Switzerland), shown in Fig. 1 ▶. The robot serves to move samples (currently up to 60) from a tray to the measurement stage in the experimental hutch. It is integrated into the beamline environment, so that applications can interact with the robot through EPICS channels (global variables broadcast over the network) in the same manner as they would with all other beamline components. For this integration, an interface was written using the robot’s VAL3 programming language making connections from other devices on the network to the robot *via* an EPICS Soft-IOC (controller computer) possible.

**Figure 2:**
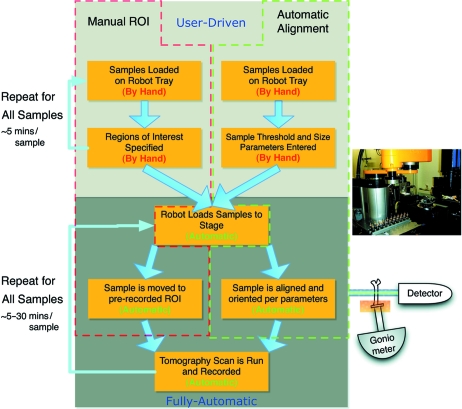
The flow of an experiment using the automated systems at TOMCAT. The two modes of operation Manual ROI (red) and Automatic Alignment (green) are shown. The Manual ROI mode is for samples where the measurement regions must be manually defined by the user, and the sample exchange and scanning is run automatically. The Automatic Alignment mode, once the thresholds have been set, is completely automatic, requiring only that the user mounts the samples on the tray and clicks start. The system will for every sample load with the robot, align using the X-ray beam and scripts, and scan.

A user-friendly interface for sample selection, naming and positioning has been designed. The software was developed in Python and scales to any number of samples. It serves as a front-end for the beamline and provides a layer of safety against collisions. The tool was designed in a modular way to be easily adaptable to updates and changes in the beamline set-up. Additionally, a scripting language was developed to allow complex management of many samples (*e.g.* repeated measurements of a sequence of samples at fixed time intervals, turning on heating or cooling, or launching of other scripts such as automatic alignment). The flow of experiments at TOMCAT is shown in Fig. 2 ▶.

For positioning and fixing the samples on the sample stage, we designed an *ad hoc* kinematic mount. This mount uses a small magnet and three precisely manufactured balls in an equilateral triangle configuration. The magnet allows the sample to be released from a height of around 1 mm and still lock into the correct final position. The accuracy of the kinematic mount and the sample holder was measured to be 0.38 µm using a laser displacement meter (LC-2420, Keyence, Woodcliff Lake, NJ, USA) (Barendregt, 2008 ▶).

There are slots for 60 samples divided into three removable trays of 20. The samples are exchanged vertically through an overhead gripper. If samples are small enough, it may be possible to stack them on top of one another (*e.g.* in capillaries), effectively multiplying the capacity of the system by the number of samples which can be stacked in this manner.

**Figure 3:**
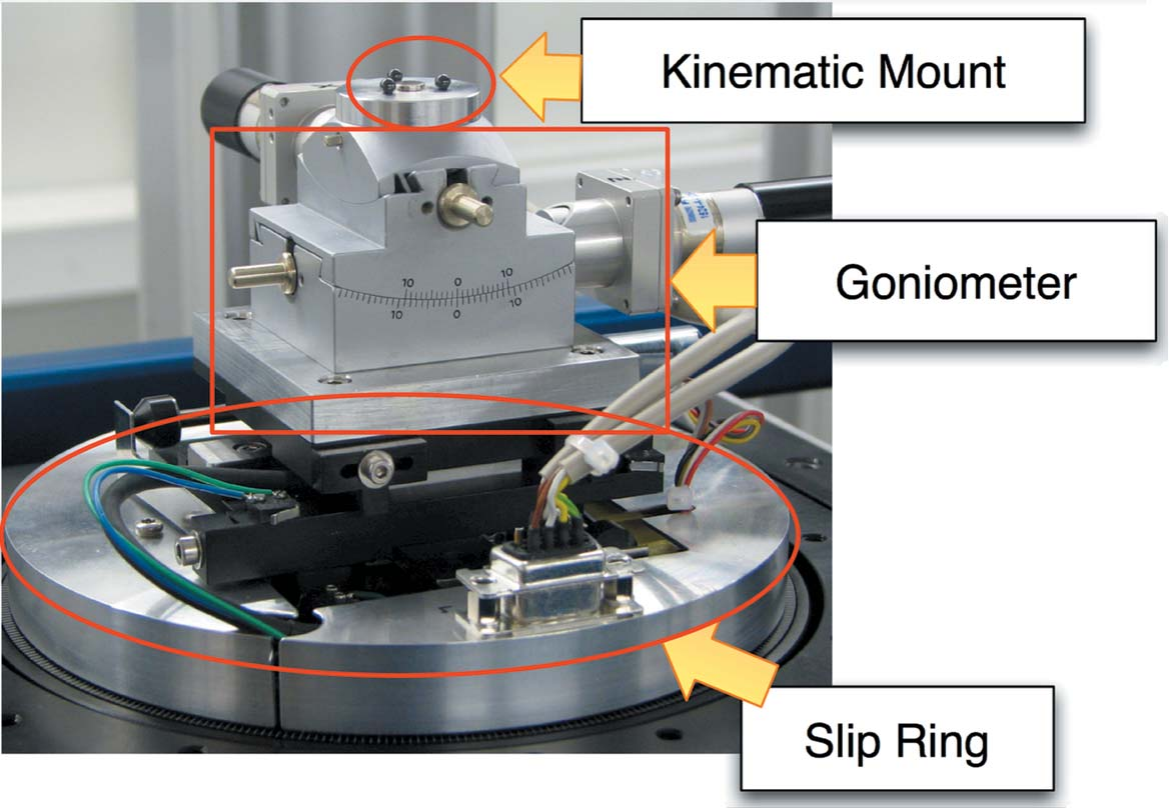
The sample stage with kinematic mount and goniometer. The slip-ring itself is not visible but is inside the labeled metal casing and carries the wires for the various stage components.

Since the task of manually fixing samples with wax or glue is time-consuming, can be difficult to ensure angular orientation, and can be tricky with sensitive samples, we utilize a gonio­meter to allow for in-beam angular re-orientation of samples. This also enables, for example, to correctly orient samples embedded in plastic, which would be difficult to do by eye. A motorized goniometer (Huber Diffraktionstechnik GmbH, Rimsting, Germany), shown in Fig. 3 ▶, was installed on the sample stage for re-orientation of the sample up to 10° in two orthogonal directions, and mounted such that the radius of rotation is aligned with the bottom of the sample. The motors are fully controlled through EPICS, and their connections are routed through the slip-ring so that the rotation of the stage is not impeded by the cabling of the goniometer.

## 3. Automatic alignment procedure for approximately cylindrical samples

For large-scale studies, automatic sample exchange is important but not sufficient. Such large-scale studies also require automatic alignment to ensure that the sample is properly centered and oriented in the field of view, maintain reproducibility, and enable truly fully automated operation. Several tools have been developed for automatic sample detection, region-of-interest location, and sample centering and angular orientation. Currently we have written a routine that successfully aligns mouse femur samples (12 × 1.1 × 1.2 mm) with a 1.5 mm field of view (10× objective, 0.74 µm pixel size). The code is, however, quite generic and, with prior knowledge of sample size, region of interest and typical sample absorption could be adapted to a variety of different types of samples. The image-processing code for segmentation and quantification is written in C++ and embedded directly in the camera control software for fastest run-time speeds. In addition, individual Python scripts use the results of the image processing to adjust the sample position.

### 3.1. Segmentation

The projection image is segmented using a simple threshold. The segmentation thresholds are quite sensitive to sample material and thickness parallel to the beam. Since this routine has been developed and tested on bone samples, we only considered three phases (three-component labeling): sample, air (low absorbing) and sample holder (high absorbing). We defined the threshold on sample absorption as a user-tunable parameter based on the material being investigated and the energy used. Consequently, air was any material that absorbed fewer X-rays than sample, sample anything that absorbed more than air but less than holder, and sample-holder anything that absorbed more than sample. While these three limits were not perfect, they sufficed to identify the boundaries of the sample in every measurement.

The largest problem source for region-of-interest identification was edge-enhancement on the wax used to mount the sample. This is caused by the combination of the high spatial coherence of the beam, finite sample size and the non-zero distance between the sample and the detector as a safety measure to prevent collisions between the stage and sensitive camera equipment. This artifact was problematic because the border of the wax was occasionally segmented as sample, when it should have been classified as air. This was greatly mediated by image cleaning by removing rows of pixels where less than 5% of the pixels were above threshold. The alignment procedures have so far only been used on bone samples, but all of the parameters involved can be adjusted to work on samples with a variety of different material compositions and shape. Specifically the threshold values can be fully adjusted to enable measurements at different energies and using materials ranging from organic compounds to metals.

### 3.2. Quantification of sample position and angular orientation

Once the image has been segmented, several parameters about the samples position can be extracted. We define a coordinate system in pixels with the origin located at the center of the image. The calibration of effective pixel sizes (pix_*x*_, pix_*y*_) in units of mm pixel^−1^ was carried out using cross-correlation of X-ray projection images and a single cone-shaped tip being moved a fixed distance by the high-precision stage motors (<1 µm). For the parameter extraction, the image is processed row by row. For each row (*y*-position) the following parameters are determined,
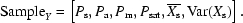
The percentages (*P*_s_, *P*_a_, *P*_m_) correspond to the ratio of pixels in the given line that were segmented as sample, air and metal, respectively. *P*_sat_ is the percentage of pixels that are saturated. 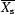 is equal to the mean *x*-value for the pixels classified as sample, and Var(*X*_s_) is the variance of the *x*-value for these pixels.

**Figure 4:**
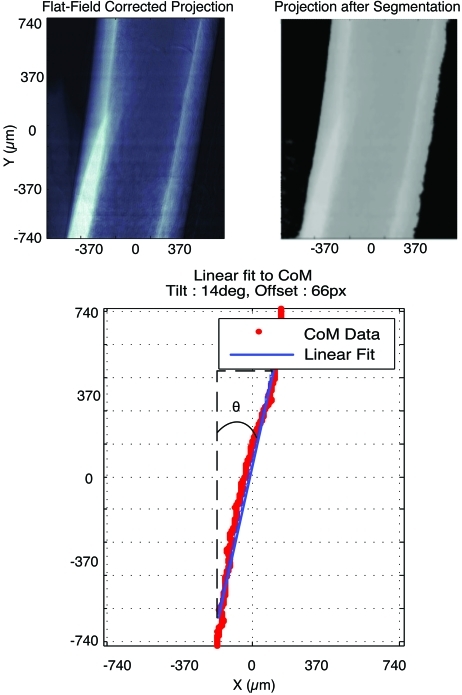
An example of the image-processing steps taken to perform the alignment on a femur at 1.4 µm pixel size. Units are given in micrometers. The upper-left image is the flat-field corrected projection, with white representing higher absorbing regions. The upper-right frame is the image after segmentation is performed using a fixed threshold. There is no metal in this image so only the two phases are visible: sample (gray) and air (black). The lower frame shows the points for the line-by-line center of mass (CoM) calculation (red points) as well as the curve fit (blue line).

To determine the sample tilt and offset from center, a line is fit through the 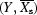 set of points,
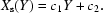
The slope *c*_1_ can then be turned into the angle of deviation from vertical alignment and the *c*_2_ value can be directly used as the horizontal offset of the image and, when scaled by the pixel size, given as a movement instruction to the stage. The correction and resulting fit can be seen on the sample in Fig. 4 ▶.

Additionally, for each projection image the highest and lowest point where *P*_s_ > 0.05 are saved as *Y*_max_ and *Y*_min_, respectively. This information can then be used to determine whether the top or bottom edge of the sample has been reached.

### 3.3. Alignment

The alignment is performed through a Python script that reads the values returned by the camera server *via* EPICS and sends instructions to the stage motors. Currently there is a library of simple commands that automatically takes the vertical rotation of the stage into account (the *X* and *Z* sample motors and goniometers are mounted on top of the rotation stage).

**Figure 5:**
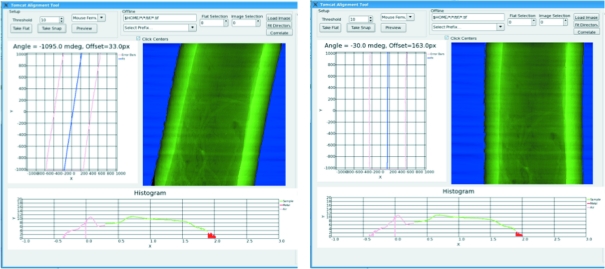
View of the user GUI before (left) and after (right) alignment. The two images show the GUI used to fine-tune the threshold and alignment parameters. The segmented image as well as a histogram and the computed values for the offset and tilt are shown.

In practice, the sample is aligned using a feedback process with the result of the aforementioned fit until the position of the object is within a user-specified tolerance. The sample is translated by the offset *c*_2_ and then tilted with the goniometer by the angle θ_s_. For a tomographic scan where the stage itself has already been aligned, it is sufficient to align the sample at 0° and 90° (frontal and sagittal planes, respectively). A before-and-after alignment picture of a femur can be seen in Fig. 5 ▶.

The alignment could theoretically be run in a single-step open-loop manner; however, there are several sources of error that could cumulatively cause a problem. While the stage has been calibrated, the local angle of deviation does not always correspond to the average or global angle for the sample. Thus it cannot be predicted how much the sample will move as the angle is adjusted.

Furthermore, with feedback, the system is not sensitive to small errors in the calibration of pixel size (the zoom provided by the lenses is not exactly 1, 2.5, 4, 10 and 20×). Although these parameters could be calculated or estimated, the feedback process usually converges after two iterations. Therefore, this calculation would not significantly improve performance.

### 3.4. User interface

The entire alignment process can be viewed and tweaked using a Python-based GUI, shown in Fig. 5 ▶. This allows for the tuning of specific parameters such as the threshold, the minimum percentage of pixels to keep, and additional settings. The GUI operates in real time providing fully processed images in less than 1 s, making it useful for experiments with non-static samples such as heating. Finally, the GUI allows for presets to be saved and loaded so once the parameters are found for a specific sample/energy combination they do not need to be determined again.

## 4. Sequencer

In order to take advantage of the previously described automation system for true hands-free operation, we have developed a sequencer application. This tool orchestrates the two earlier programs and allows the user to write pseudo-code for the steps to be done during an experiment. The sequencer is tightly integrated in the beamline database allowing the user to easily use samples and regions of interest, which have been saved there.

### 4.1. Simple linear experiments

The most common use of the sequencer is for simple experiments where a number of probes have been aligned and one or more regions of interest saved. In this case the sequencer just serves to change the samples, move the sample to the region of interest, and run the scan. In the case of automatic sample alignment, the sequencer executes the alignment tool at the proper time.

### 4.2. Time-lapse experiments

For time-lapse experiments the sequencer allows for a given sequence of samples to be measured repeatedly to monitor changes in a fixed region of interest during processes such as drying, compression and heating. The sequencer also allows for control of other devices.

## 5. Results

The robot is currently in full operation at the TOMCAT beamline and has already been tested on cement, bone, fossils, rock and brain samples. The system has proved to be safe enough for one-of-a-kind samples such as fossils, and stable enough for overnight or weekend-long beam times. A few example studies have been taken to demonstrate practical applications of the features developed. The reproducibility and ability to study the time evolution of samples is shown in a cement dehydration study (§5.1). The ability to record multiple regions of interest and then run scans for many hours without intervention are shown in a study on mouse brains (§5.2) and meteorite samples (§5.3). Finally, the ability of the system to conduct measurements completely autonomously from mounting to alignment and scanning is demonstrated in the mouse femur study (§5.4).

### 5.1. Time evolution experiments: repeatability

The goal of this experiment was to study the aging process of cement paste in three dimensions. The set-up consisted of three samples, which were consecutively scanned over the course of 12 h (one measurement every 30 min). The scans were conducted by taking 1001 projections with a 200 ms exposure time at an energy of 14 keV and pixel size of 0.74 µm. In addition to the automatization of the experiment (no human intervention for 12 h), the use of the sample-exchange system enabled the repositioning of samples on the stage at regular time intervals with an extremely high degree of reproducibility (<2 µm). This is a very desirable feature because usually three-dimensional image registration is quite complicated and requires custom sample-specific implementations. Furthermore, in evolving samples this can be even more difficult since distinct features used for registration may disappear during sample evolution.

### 5.2. Multiple region-of-interest scanning

**Figure 6:**
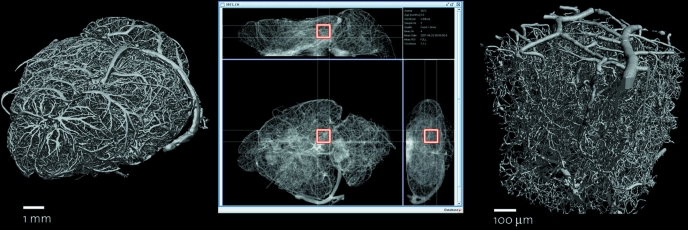
(Left) Three-dimensional image of the mouse brain vasculature imaged with 12 µm voxel size. (Middle) A single region of interest (red box) selected within the left posterior hippocampus. (Right) Three-dimensional rendering of the region measured with a 1 µm voxel size.

For studies of the microvasculature in mouse brains it is important to have an overview scan of the entire brain at low resolution (Fig. 6 ▶) and then higher-resolution regions of interest in biologically relevant areas (Heinzer *et al.*, 2008 ▶). The overview scan is important for understanding the holistic connectivity of the vascular network and the higher resolution is required to visualize the smaller capillary networks. For each sample between five and ten regions of interest are measured. Data acquisition for each region of interest takes about 15 min. For this set-up the samples are manually aligned in reference to a fixed pin-based coordinate system so that the higher- and lower-resolution scans are comparable. This alignment is then used as the basis for accessing the predefined regions. The initial manual alignment takes around 5–10 min per sample. When the system is loaded with 60 aligned samples, it is then theoretically capable of running unattended for up to 225 h. In practical tests the set-up was successfully run for 53 h without user intervention. During this beam time over 200 tomographic scans were made with a voxel size of 0.74 µm at an energy of 13.5 keV with 1001 projections and 87 ms exposure time. Given that the average beam time granted is 48 h, the system is more than capable of handling the standard experiment.

### 5.3. Stacked samples in a capillary

Using the region-of-interest selection and saving tool, it is possible to make multiple scans per sample. This is ideal for small samples stacked vertically in a capillary. On a set of meteorite samples, we ran 127 high-resolution (350 nm pixel size) scans at an energy of 17.5 keV with 1501 projections of 300 ms each, over the course of 27 h. The regions of interest were manually selected at the beginning, requiring 3 h of user time.

### 5.4. High-throughput studies

The final goal of the system is to be able to run genome-scale studies and therefore handle a very large number of samples (up to several thousand). For these studies, sample position and orientation must be consistent, so that results for different samples can be meaningfully compared. Specifically, a large-scale project involving the investigation of ultrastructure in mice femurs provided the ideal sample type (approximately cylindrical) to validate the automatic alignment. For the femur samples used in the study, the region of interest was at 56% of the length of the bone as empirically determined in previous studies (Schneider *et al.*, 2007 ▶; Kohler *et al.*, 2007 ▶). In general, it is known that when comparing samples for the investigation of morphological properties of bone, a relative percentage of the femur length is biologically much more meaningful than a fixed physical metric distance. In order to use a percentage of the femur length, this length must be measured. This is done by taking the parameters (*Y*_max_, *Y*_min_) calculated automatically for each image. (*Y*_max_, *Y*_min_) are around 1024 and −1024, respectively, when the bone vertically fills the field of view (2048 pixels). The sample is then moved using the *Y*-stage motor in half-image steps until *Y*_max_ is equal to 0 (the top edge is in the middle of the field of view) and then in the opposite direction until *Y*_min_ is equal to 0 (the bottom edge is in the middle of the field of view). The length of the bone is then calculated by taking the difference of the two absolute *Y*-stage motor positions. In this manner the accuracy of the positioning lies almost entirely in the accuracy of the stage instead of being also largely sensitive to the estimated pixel size.

#### 5.4.1. Automatic alignment precision

**Figure 7:**
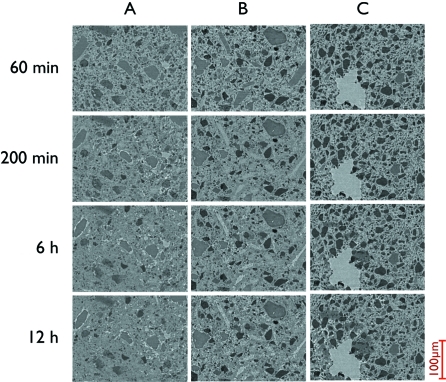
A single slice taken from separate measurements of three different cement-filled capillaries over the course of 12 h. During this time the three different samples were automatically loaded, scanned and unloaded with the robot. The drift between consecutive images was calculated using cross-correlation to be on average 1.50 µm. Images courtesy of Gastaldi *et al.* (2011 ▶).

The accuracy and precision of the alignment procedure has been experimentally verified using four different samples subsequently loaded and unloaded five times each. Each of these samples was loaded and moved to a random position up to one field of view away from the aligned position. The alignment script was run and the positions of the *x*, *y* and *z* stage motors were read after alignment. For this purpose the stage was assumed to be a reliable measure of absolute position (as shown in Fig. 7 ▶ the accuracy is better than 2 µm). If a sample was aligned identically every time, the values would be the same. Therefore the standard deviation of the stage position values is a good metric of alignment precision.

The variation in the *x*, *y*, *z* positions of the sample at alignment was in three of the four cases less than 15 µm, corresponding to 21 of 2048 pixels or 1% of the field of view. On the fourth sample the results were considerably worse (still tolerable) with a precision of the alignment being ∼180 µm, as it was larger than the field of view so the algorithm had to estimate the center and tilt from incomplete information (Table 1 ▶).

#### 5.4.2. Experiment

**Figure 8:**
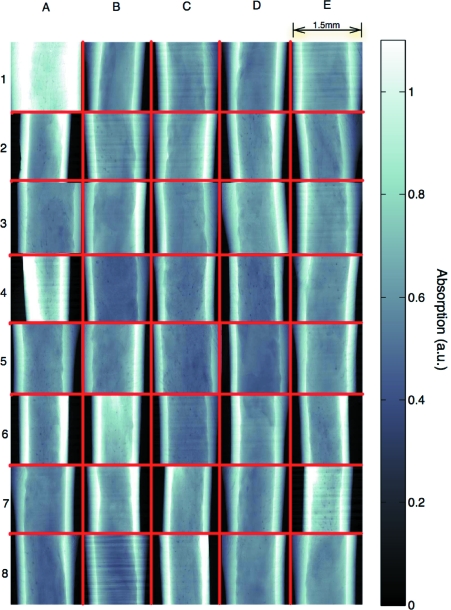
Projections of 40 aligned bones. The images are organized in a 5 × 8 grid (red) and are flat-field and logarithmically corrected. Brighter intensity represents more absorption. Some of the samples are slightly larger than the field of view, but all are straight and centered within the 10 µm tolerance. Notice in images E2 and D3 that the bone appears to be slightly tilted. This is due to the anisotropic shape of the bone and in these cases the alignment of the edges provides a different result than the alignment of the center of mass based on absorption. The center of mass alignment is advantageous as it maximizes the volume of bone that is within the field of view.

The operation of fully automatic alignment has been validated on over 1000 samples (Fig. 8 ▶) from the study. This means that the samples were manually placed on the sample holders using wax (quickly, without microscope, taking 20 s per sample) and loaded onto the tray. The sample-exchange robot proceeded to take each sample, mount it on the stage, find the region of interest, center and align it, finally scan it and return it to the tray. With a scan time of 7 min (energy 17.5 keV, 1501 projections, 160 ms exposure time, and 1.48 µm pixel size, with two-fold binning), the system aligned *and* measured the femurs over the course of five beam times with a single operator, at a final rate of around 4.4 samples per hour. Therefore the total time spent for changing and aligning the sample was 6 min and 15 s. In direct comparison with manual operation the system represents a marked improvement. An experienced user requires about 10 min to manually align the femurs during mounting and around 5 min for security checks and centering the stage.

## 6. Conclusion

The automatization tools developed at the TOMCAT beamline greatly simplify the arduous task of tomography on many samples. The system increases throughput, while decreasing the likelihood of human error and user-caused damage at the beamline. Furthermore, the system protects the sensitive beamline equipment from the effects caused by excessive manual handling. Manpower requirements for measuring many samples are greatly reduced since once the initial alignments and regions of interest are entered the system requires no further administration, allowing users to begin on-site data analysis. In summary, the implementation of these new automatization tools results in faster, less variable and more efficient beam times at TOMCAT.

For samples where the automatic alignment (§3) is used, the benefit of the system is even greater since the user simply needs to load the samples on a tray, assign them names in the user interface, and click start (for femur samples approximately 30 min of work for 60 samples). From this point forward the system carries out all procedures without user intervention. The system uses a robot and intelligent algorithms to load, find, align and scan the sample in a highly precise, stable and reproducible manner. This is the first time that such an instrument has been developed for synchrotron-based tomographic microscopy applications.

The potential to realise both larger scale and time-evolution experiments is the true strength of such an automated system. Additionally, the system, by handling logistical issues involved in conducting a large and reproducible study, enables research to be science-driven. To this end we are developing algorithms to work with more complicated samples and a storage system so that environmentally sensitive samples can be measured.

We believe that our developments represent a powerful step forward in automation at beamlines and a crucial development to enable genome-scale studies in tomographic microscopy. The combination of robotics, computer-controlled measurement and software automation has reduced significantly the amount of man hours involved in making tomography measurements.

## Figures and Tables

**Table 1:** Results from the test on accuracy and reproducibility of the alignment routines on samples of varying sizes The samples were taken to represent the full range of femur bones to be used in the study selected by length and width with the length varying from 8.8 mm to 13.8 mm. The length as shown is the calculated length using the difference between the motor positions at the top and bottom of the sample. *X*, *Y*, *Z* are the motor positions when the sample is in the measurement region.

Sample	Number of repetitions	Total time (s)	Length (mm)	*X* (µm)	*Y* (µm)	*Z* (µm)
1	8	485 ± 22	13.8 ± 0.44	−1053 ± 188.5	−1313 ± 187.6	663 ± 35.9
2	6	497 ± 72	12.7 ± 0.17	−1547 ± 7.1	−610 ± 100.5	549 ± 8.5
3	5	423 ± 5	10.9 ± 0.08	1164 ± 10.9	−157 ± 44.8	1164.4 ± 10.9
4	7	380 ± 10	8.8 ± 0.17	−912 ± 14.0	1104 ± 100.5	−16 ± 3.3
